# Circular RNA FLNA acts as a sponge of miR-486-3p in promoting lung cancer progression via regulating XRCC1 and CYP1A1

**DOI:** 10.1038/s41417-021-00293-w

**Published:** 2021-01-26

**Authors:** Jiongwei Pan, Gang Huang, Zhangyong Yin, Xiaoping Cai, Enhui Gong, Yuling Li, Cunlai Xu, Zaiting Ye, Zhuo Cao, Wei Cheng

**Affiliations:** 1 Department of Respiratory, the Sixth Affiliated Hospital of Wenzhou Medical University/Lishui People’s Hospitlal, Lishui, Zhejiang 323000 China; 2 Department of Chinese Medicine, the Sixth Affiliated Hospital of Wenzhou Medical University/Lishui People’s Hospitlal, Lishui, Zhejiang 323000 China; 3 Department of Radiology, the Sixth Affiliated Hospital of Wenzhou Medical University/Lishui People’s Hospitlal, Lishui, Zhejiang 323000 China; 4 The Sixth Affiliated Hospital of Wenzhou Medical University; Longquan Branch, Lishui People’s Hospitlal, Lishui, China; 5 Department of Anesthesiology, the Affiliated Hospital of Xuzhou Medical University, Jiangsu Province Key Laboratory of Anesthesiology and Center for Pain Research and Treatment, Xuzhou, Jiangsu 221002 China

**Keywords:** Cancer, Genetics

## Abstract

Significantly high-expressed circFLNA has been found in various cancer cell lines, but not in lung cancer. Therefore, this study aimed to explore the role of circFLNA in the progression of lung cancer. The target gene of circFLNA was determined by bioinformatics and luciferase reporter assay. Viability, proliferation, migration, and invasion of the transfected cells were detected by CCK-8, colony formation, wound-healing, and transwell assays, respectively. A mouse subcutaneous xenotransplanted tumor model was established, and the expressions of circFLNA, miR-486-3p, XRCC1, CYP1A1, and related genes in the cancer cells and tissues were detected by RT-qPCR, Western blot, or immunohistochemistry. The current study found that miR-486-3p was low-expressed in lung cancer. MiR-486-3p, which has been found to target XRCC1 and CYP1A1, was regulated by circFLNA. CircFLNA was located in the cytoplasm and had a high expression in lung cancer cells. Cancer cell viability, proliferation, migration, and invasion were promoted by overexpressed circFLNA, XRCC1, and CYP1A1 but inhibited by miR-486-3p mimic and circFLNA knockdown. The weight of the xenotransplanted tumor was increased by circFLNA overexpression yet reduced by miR-486-3p mimic. Furthermore, miR-486-3p mimic reversed the effect of circFLNA overexpression on promoting lung cancer cells and tumors and regulating the expressions of miR-486-3p, XRCC1, CYP1A1, and metastasis/apoptosis/proliferation-related factors. However, overexpressed XRCC1 and CYP1A1 reversed the inhibitory effect of miR-486-3p mimic on cancer cells and tumors. In conclusion, circFLNA acted as a sponge of miR-486-3p to promote the proliferation, migration, and invasion of lung cancer cells in vitro and in vivo by regulating XRCC1 and CYP1A1.

## Introduction

Lung cancer is the most malignant tumor worldwide and the leading cause of cancer-related deaths with the highest mortality [1, 2]. According to statistics, there are around over 1.8 million new cases and more than 1.6 million deaths resulted from lung cancer per year [2]. The insidious symptoms of early lung cancer lead to the loss of surgical opportunities at initial diagnosis, and therefore, chemotherapy alone or in combination with radiotherapy has been adopted as the main treatment method for patients with advanced lung cancer [3]. Currently, the 5-year survival rate of lung cancer patients has remained as low as 16% [4]. The occurrence and development of lung cancer are closely related to the abnormal expressions of multiple genes, such as oncogenes, tumor suppressor genes, and growth-related genes [2–4]. Hence, the identification of cancer-related genes is highly beneficial to developing novel therapeutic targets for the prevention and treatment of lung cancer.

Circular RNAs (circRNAs), a class of endogenous non-coding RNAs, are characterized by covalently closed-loop structures without 5′ and 3′ ends contained in the linear RNAs [5]. Evidence has increasingly proved that circRNAs play an important role in carcinogenesis and cancer development [6]. For instance, circSLC8A1 could inhibit the progression of bladder cancer [5]; circ100338 enhances liver cancer metastasis [7]; and circ104433 regulates the growth of gastric cancer cells [8]. In addition, circFLNA (circRNA ID: hsa_circ_0092012), a product of splicing exon-9 to exon-15 of the FLNA gene, has been recently reported to be up-regulated in human laryngeal and oral squamous cell carcinoma. However, there was a lack of knowledge of the expression level and role of circFLNA in lung cancer.

MicroRNAs (miRNAs) are a family of endogenous, small (19-25 nucleotides) non-coding single-stranded RNA molecules with crucial functions in many biological processes, including in cell proliferation, apoptosis, and metastasis [9–11]. miR-486-3p is associated with anaplastic lymphoma kinase translocation in lung adenocarcinoma [12], and prediction from microarray analysis showed a low expression of miR-486-3p in lung cancer [13]. However, the role of miR-486-3p in lung cancer remains unknown. Previously, it has been recognized that circRNAs may function through sponging some miRNAs and regulating cancer progression [14]. The regulatory effect of circSLC8A1 on bladder cancer is mediated by sponging miR-130b [5]; circ104433 acts as a sponge of miR-497-5p in regulating the growth of gastric cancer cells [8]; circ0000502 also sponges miR-124 to regulate liver cancer metastasis and apoptosis [15]. Nevertheless, whether circFLNA harbors miR-486-3p with a regulatory effect on lung cancer cells is yet to be revealed.

Therefore, the purpose of this study was to explore the roles of circFLNA and miR-486-3p in lung cancer and further investigate whether circFLNA sponges miR-486-3p in lung cancer.

## Methods

### Ethics statement

All animal experiments were performed in accordance with the guidelines of the China Council on Animal Care and Use. This study was approved by the Committee of Experimental Animals of the Sixth Affiliated Hospital of Wenzhou Medical University/Lishui People’s Hospitlal. Every possible effort has been devoted to minimize pain and discomfort to the animals. The animal experiments were performed in the Sixth Affiliated Hospital of Wenzhou Medical University/Lishui People’s Hospitlal.

### Cell culture

Normal human bronchial epithelial cell line BEAS2B (CRL-9609) and human lung cell lines NCI-H520 (HTB-182), SK-MES-1 (HTB-58), H1299 (CRL-5803), A549 (CCL-185), and H23 (CRL-5800) were obtained from the American Type Culture Collection (Rockville, MD, USA). Normal human bronchial epithelial cell line 16HBE (ml028972) was purchased from Mibio (Shanghai, China). Human lung cell line PC-9 (BNCC341852) was provided by BeNa Culture Collection (Beijing, China). All cells were cultured in RPMI 1640 medium (C11875500BT, Gbico, MA, USA) containing 10% fetal bovine serum (FBS; 10437010, Gbico) and incubated at 37 °C in a humidified atmosphere with 5% CO_2_.

### Transfection

Small interfering RNA for circFLNA (sicircFLNA), the circFLNA overexpression plasmids, the XRCC1 overexpression plasmids, and the CYP1A1 overexpression plasmids were synthesized by GenePharma (Shanghai, China). The circFLNA overexpression plasmids were ligated into the pLVX-cir vector, whereas the XRCC1 and CYP1A1 overexpression plasmids were ligated into pcDNA3.1. The sequence of sicircFLNA was 5′-GCCAGCUCCCUGAAGGGGCTT-3′. The primer sequences were as follows: circFLNA-F: 5′-GCTTGGCCAACAGTGACAGTGTAGG-3′, circFLNA-R: 5′- CAGCTACCAGCCCACCATGGAG-3′; CYP1A1-F: 5′-CAGTGAAGAGGTGTAGCCGCT-3′, CYP1A1-R: 5′- TAGGAGTCTTGTCTCATGCCT-3′; XRCC1-F: 5′-ACTGCTGGAACCTGGCCCTGC -3′, XRCC1-R: 5′- GCAAACCCCGAGGAGAAGGCA -3′. MiR-486-3p mimic (miR10004762-1-5; 5′- UAGGACAUGACUCGACGGGGC-3′), mimic control (miR1N0000002-1-5; 5′- UUCUCCGAACGUGUCACGUUU-3′), negative control for siRNA (siNC; siN0000002-1-5), and negative control for overexpression (NC; lnc6N0000002-1-10) were all obtained from RIBOBIO (Guangzhou, China).

The products were diluted with RNase-free H_2_O (ST876, Beyotime, Shanghai, China) and stored at -20 °C for later use. Before transfection, lung cancer cells were plated into six-well plates in 2 ml of complete medium at a density of 1.0 × 10^6^ cells/well. After the cells were cultured overnight till 20–30% confluence, 100 μl of medium was added to dilute 2 μg of plasmids and siRNA, and 3 μl of lipofectamine 2000 (11668-019, Invitrogen, MA, USA) was added to another 100 μl of medium. Then the two media were mixed together for a 15-min incubation at room temperature. Finally, the mixed solution was added into the cells of each well, followed by the addition of 1.8 ml of medium for an additional 48-h cell incubation.

### Luciferase reporter assay

The fragments of the 3′ UTRs of wild type circFLNA (circFLNA-WT, 5′-GTCCAGGACAATGAAGGCTGCCCTG-3′) and mutant circFLNA (circFLNA-MUT, 5′-GGTAAGGGTAACTGAGTAGTAATTA-3′), the 3′ UTRs of wide-type XRCC1 (XRCC1-WT, 5′-ATCTGACCTCAGCACTGCCCCT-3′) and mutant XRCC1 (XRCC1-MUT, 5′-ATCTGACCTCAGCAAGTTAATT-3′), and the 3′ UTRs of wide-type CYP1A1 (CYP1A1-WT, 5′-CCTAAGGGATCCTGCCTGCCCCT-3′), and mutant CYP1A1 (CYP1A1-MUT, 5′-ACCTAAGGGATCCTGCCTCTTTAAT-3′) containing binding sites for miR-486-3p were inserted into pmirGLO luciferase Vectors (E1330, Promega, CA, USA). SK-MES-1 and A549G cells were placed into 48-well plates, with each well containing 3.0 × 10^4^ cells in 300 μl of complete medium. After growing overnight, these target or non-target vectors were co-transfected with miR-486-3p inhibitor (miR20004762-1-5, RIBOBIO) or mimic into SK-MES-1 and A549G cells using lipofectamine 2000. After 48 h of cell transfection, the cells were then subjected to perform Dual-Luciferase Reporter Assay (Promega). Luciferase activity of the cells was determined with a GloMax fluorescence reader (Promega).

### CCK-8 assay

CCK-8 (KGA317s-3000, KeyGEN BioTech, Jiangsu, China) was used to detect cell viability. After transfection, SK-MES-1 and A549G cells were laid into 96-well plates, with each well containing 1.0 × 10^4^ cells in 100 μl of complete medium. After growing for 24, 48, or 72 h, the cells were incubated with 10 μl of CCK-8 reagent for 4 h. Finally, the absorbance of each well was measured at 450 nm using a microplate reader (Infinite M200 PRO, Tecan Austria GmbH, Austria).

### Colony formation assay

After transfection, SK-MES-1 and A549 cells at a density of 1000 per group were seeded into a 6-well plate and cultured for 14 days for colony formation. Then the colonies formed were fixed with 4% formaldehyde (P804536, Macklin, Shanghai, China) for 10 min, followed by visualization treatment with 0.3% crystal violet (C0121, Beyotime) for 15 min. After removing excessive crystal violet by rinsing the plate in PBS, the visible colonies were counted and analyzed using Image J software (Version 1.8.0).

### Wound-healing assay

After transfection, SK-MES-1 and A549 cells were placed into six-well plates at 3.5 × 10^5^ cells/well in 2 ml of complete medium and cultured till 95% of cell confluence. Then, a vertical wound in each well was created with a 20-μl pipette tip, and a medium without FBS was added into each well. Images of each well were collected at 0 and 48 h under a phase-contrast optical microscope (Axio Lab.A1 pol; Leica, Solms, Germany). Image J software (Version 1.8.0) was used to analyze the images.

### Transwell assay

Transwell cell culture chambers were pre-coated with Matrigel (354234, Corning Life Sciences, NY, USA) and placed into a 24-well plate. The transfected cells were diluted into 2 × 10^5^ cells/well and pipetted into the chambers containing a suspension solution with 0.2 ml of FBS-free medium, and the corresponding complete medium was added into the lower chamber. After the cells were incubated for 48 h, the upper-side of the polycarbonate membrane was wiped off, leaving the underside of the membrane containing invaded cells. After that, the cells were stained with crystal violet for 15 min at room temperature. Finally, cell number was counted from three randomly chosen areas on each membrane (×250) under a phase-contrast optical microscope (Axio Lab.A1 pol; Leica, Solms, Germany). Image J software (Version 1.8.0) was applied to analyze the images.

### Animals and subcutaneous xenograft

In this study, 24 six-week-old male BALB/c nude mice (weight: 20–22 g) were obtained from SLAC Laboratory Animal Technology (Shanghai, China). All the experimental animals were fed in the same animal feeding unit and maintained under a 12-h dark/light cycle in an SPF-controlled environment. The animals were randomly divided into four groups (*n* = 6) as follows: NC + MC, circ+MC, NC + M, and circ+M groups.

For the NC + MC group, three mice were subcutaneously injected with 2 × 10^6^ SK-MES-1 cells in the right flank area and the other three mice were injected with 2 × 10^6^ A549 cells in the same way. All cells had been co-transfected with overexpression negative control and mimic control. For the circ+MC group, three mice were subcutaneously injected with 2 × 10^6^ SK-MES-1 cells in the right flank area and the other three mice were similarly injected with 2 × 10^6^ A549 cells. All the cells had been co-transfected with overexpression circFLNA plasmids and mimic control. For the NC + M group, three mice were subcutaneously injected with 2 × 10^6^ SK-MES-1 cells in the right flank area and the other three mice were similarly injected with 2 × 10^6^ A549 cells. All the cells had been co-transfected with overexpression negative control and miR-486-3p mimic. For the circ+M group, three mice were subcutaneously injected with 2 × 10^6^ SK-MES-1 cells in the right flank area and the other three mice were similarly injected with 2 × 10^6^ A549 cells. All the cells had been co-transfected with circFLNA plasmids and miR-486-3p mimic. Four weeks after injection, the mice were anesthetized with 2% sodium pentobarbital (50 mg/kg) (B005, Jiancheng, Nanjing) and killed by cervical dislocation. Then, the tumor tissues were harvested and weighed. In addition, tumor volume changes in each group were recorded at day 5, 10, 15, 20, 25, and 28.

### RNA extraction

MiRNAs were extracted from the tumor tissues and cultured cell lines using a miRcute miRNA Isolation Kit (TianGEN, Beijing, China). In brief, the tissue samples were ground in liquid nitrogen using a grinding rod in a 1.5-ml centrifugal tube containing lysis buffer, followed by collection into a 1.5-ml centrifugal tube and addition of lysis buffer. Then, 200 μl of chloroform (C805334, Macklin) was added to the cells and shaken for 1 min. After resting for 5 min at room temperature, the cells were centrifuged for 20 min (13,400 × *g*) and collected using miRNA solution into a new 1.5 ml tube. The cells were subsequently added with 75% ethanol and further centrifuged for 15 min (13,400 × *g*). The miRNA precipitation was diluted with RNase-free H_2_O.

Cytoplasmic and nuclear RNAs were extracted from the cultured cell lines using a Cytoplasmic & Nuclear RNA Purification Kit (37400, NORGEN BIOTEK, Thorold, Canada). In brief, 3 × 10^6^ cells were lysed with Lysis Buffer J in a 1.5-ml centrifuge tube and centrifuged for 10 min (14,000 × *g*). Then the supernatant was collected for the extraction of cytoplasmic RNA and the precipitation was collected for nuclear RNA extraction. The supernatant and the precipitation were mixed with 200 μl of Buffer SK and 400 μl of Buffer SK for 10 s, respectively, and subsequently, the mixtures were separately added with 200 μl of 100% ethanol (E111991, Aladdin, Shanghai, China). Then each mixture was added into a rotating column assembled with a collecting tube, and centrifuged for 1 min (3500 × *g*). Afterwards, the rotating columns were further assembled with new elution tubes. Finally, 50 μl of Elution Buffer E was added into the rotating columns and centrifuged for 1 min (14,000 × *g*) to collect cytoplasmic and nuclear RNAs.

Total RNAs containing mRNA and circRNA were also extracted from the tumor tissues and cultured cell lines. Briefly, the tissues and cells were lysed using TRIzol (15596, Invitrogen) and collected into a new 1.5 ml centrifuge tube. Then, chloroform was added into the tube and centrifuged for 20 min (14,000 × *g*). The supernatant was collected, added with an equal volume of isopropanol (H822173, Macklin), and further centrifuged for 5 min (14,000 × *g*). Finally, the RNA precipitation was diluted using RNase-free H_2_O.

### RNase R treatment

RNAs (2.5 μg) extracted from SK-MES-1 and A549 cells were incubated with 10U RNase R reagent (M1228, BioVision Incorporated, CA, USA) at 37 °C for 30 min for later use in RT-qPCR.

### RT-qPCR

After the extraction of miRNAs or total RNAs from the tumor tissues and cultured cell lines, a PrimeScript RT kit (RR037A, Takara, Dalian, China) was used to reverse-transcribe the RNAs into cDNAs (all the RNAs used for circRNA expression detection had been previously incubated with RNase R before reverse-transcription). Gene expression was detected by RT-qPCR assay using a Verso 1-step RT-qPCR Kit (A15300, Thermo Scientific, MA, USA) in ABI 7500 Fast Real-Time PCR System (Applied Biosystems, CA, USA), and the condition of RT-qPCR was set as follows: at 95 °C for 30 s, at 60 °C for 30 s, and 45 cycles of at 60 °C for 30 s. RNA was quantified by the 2^−△△CT^ method. All primer sequences are shown in Table 1.

**Table 1:** RT-qPCR primers.

Target gene	Forward primers, 5′–3′	Reverse primers, 5′–3′
miR-486-3p	GGTAGAAAAAGCAACCACGAAG	ACATAAACCTCTGTCTGTGAGTGC
circFLNA	CCAGCTGAGGCTCTACCGTGCC	GAGGCGTCAGCATCCCCAACAG
FLNA	AATGTGACGACAAGGGCGAC	AGCACGTGAACGGCATACTC
E-Cadherin	CGAGAGCTACACGTTCACGG	GGGTGTCGAGGGAAAAATAGG
N-Cadherin	TCAGGCGTCTGTAGAGGCTT	ATGCACATCCTTCGATAAGACTG
Vimentin	GACGCCATCAACACCGAGTT	CTTTGTCGTTGGTTAGCTGGT
WNK2	CGCTTCCTCAAGTTCGACATC	TGGACTCCCAGAAGTCGTAGA
CYP1A1	TCGGCCACGGAGTTTCTTC	GGTCAGCATGTGCCCAATCA
PAX5	ACTTGCTCATCAAGGTGTCAG	TCCTCCAATTACCCCAGGCTT
XRCC1	TCAAGGCAGACACTTACCGAA	TCCAACTGTAGGACCACAGAG
TGM3	ATGGCTGCTCTAGGAGTCCAG	GTTTTGGCCTCTCCGCAAGAT
Bcl-2	GGTGGGGTCATGTGTGTGG	CGGTTCAGGTACTCAGTCATCC
Bax	CCCGAGAGGTCTTTTTCCGAG	CCAGCCCATGATGGTTCTGAT
U6	CTCGCTTCGGCAGCACA	AACGCTTCACGAATTTGCGT
GAPDH	AGGTCGGTGTGAACGGATTTG	GGGGTCGTTGATGGCAACA

Western blotting original image.

### Western blot

Total protein from the tumor tissues or cultured cell lines was isolated with RIPA lysis buffer (P0013B, Beyotime), and a BCA assay kit (23250, Pierce, MA, USA) was used to determine the total protein concentration. Total protein (25 µg) was separated in each lane on 10% SDS-PAGE gels (P0052A, Beyotime), electro-blotted and transferred to NC membranes (HTS112M, Millipore,). Then all the membranes were incubated with 5% skimmed milk for 2 h at room temperature, followed by incubation with the following primary antibodies: E-Cadherin (1:1000, ab40772, 97kD, Abcam, CA, USA), N-Cadherin (1:1000, ab18203, 130kD, Abcam), Vimentin (1:1000, ab92547, 54kD, Abcam), cleaved Caspase-3 (1:1000, ab2302, 17kD, Abcam), Bcl-2 (1:1000, ab59348, 26kD, Abcam), Bax (1:1000, ab32503, 21kD, Abcam), XRCC1 (1:1000, ab134056, 69kD,Abcam), CYP1A1 (1:1000, ab79819, 58kD, Abcam), Ki67 (1:1000, ab16667, 359kD, Abcam), PCNA (1:1000, ab92552, 29kD, Abcam), and GAPDH (1:1000, 36kD, ab8245, Abcam). The next day, the membranes were further incubated with HRP-conjugated secondary antibodies goat anti-mouse IgG secondary antibody (1:5000, ab205719, Abcam) and goat anti-rabbit IgG secondary antibody (1:5000, ab205718, Abcam) for 1 h at room temperature. Finally, SuperSignal West Pico Chemiluminescent Substrate (34078, Thermo Scientific) was applied to incubate the membranes for signal detection. Image Lab™ Software (version 3.0) was used for densitometric analysis and quantification of the Western blot data (Bio-Rad Laboratories Inc., Hercules, CA, USA).

### Immunohistochemical

The tumor tissues were harvested and embedded in paraffin (S25190, Yuanye, Shanghai, China). Then the tissues were fixed on the microtome (RM2235, Leica, Solms, Germany) and cut into 4 μm thick slices. Next, the slices were fixed on a glass slide (P105-2001, MeVid, Jiangsu, China, http://www.nt-mevid.com/ProDetail.aspx?ProId=54) and deparaffinized. Subsequently, the tissue slices were incubated with antigen repair solution (p0081, Beyotime) for 10 min at room temperature, followed by incubation with endogenous peroxidase blocker (BF06060, Biodragon, Beijing, China) for another 10 min at room temperature. Afterwards, the tissues were placed into 5% FBS to block for 1 h at room temperature, and separately incubated with XRCC1 antibody (1:300, ab134056, Abcam) and CYP1A1 antibody (1:50, ab79819, Abcam) overnight at 4 °C. A corresponding secondary antibody (G-21234, 1:500, Thermo Scientific) was then incubated with the tissues for 30 min, followed by the treatment with a DBA reagent (SFQ004, 4 A Biotech, Beijing, China) for 30 min. After the tissue slices were treated with hematoxylin (B25380, Yuanye) for 10 min, the indexes were finally observed and recorded using a phase-contrast optical microscope (Axio Lab.A1 pol; Leica, Solms, Germany). For the quantification of immunohistochemistry, five random fields from each slice were selected to calculate the ratio of the number of positive cells to the total number of cells.

### Statistical analysis

Data generated in this study were analyzed by student’s t-test and one-way ANOVA using SPSS software (version 18.0). LSD and Dunnet’s were used as post-hoc tests. Pearson analysis was used to analyze the correlation between circFLNA and miR-486-3p. Statistical data were expressed as mean ± standard deviation. All experiments were conducted three times. *P* < 0.05 was considered as statistically significant.

## Results

### MiR-486-3p was low-expressed in lung cancer tissues and cells

**Fig. 1:**
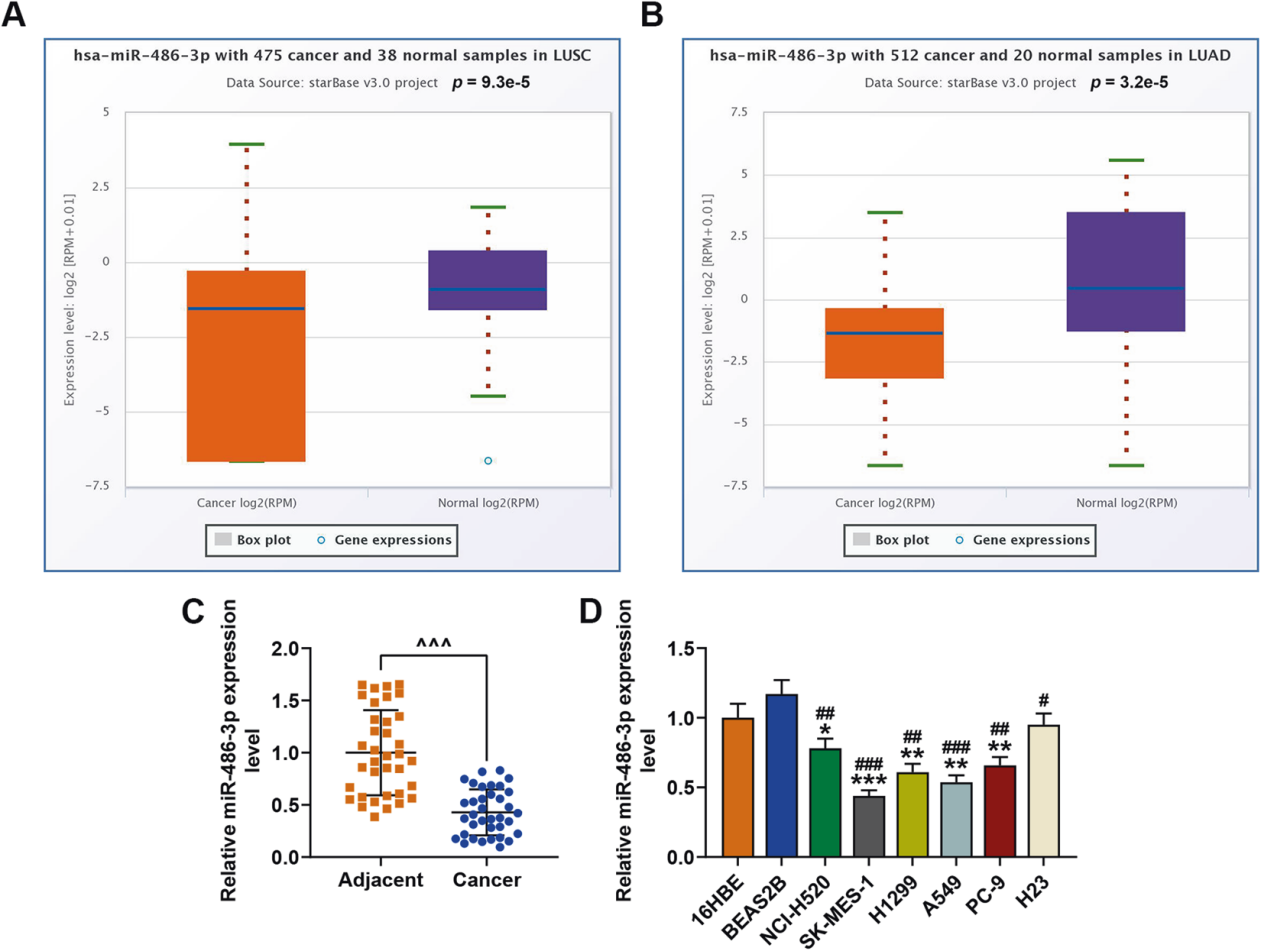
MiR-486-3p was low-expressed in lung cancer tissues and cells. **A**, **B** The expression level of miR-486-3p in lung cancer tissues and normal tissues was analyzed using starBase. **C** QRT-PCR was used to detect the miR-486-3p expression in the adjacent tissues and cancer tissues. **D** The expression level of miR-486-3p in lung cancer cell lines (NCI-H520, SK-MES-1, H1299, A549, PC-9, and H23) and normal bronchial epithelial cell lines (16HBE and BEAS2B) was determined by RT-qPCR. U6 was used as an internal control. All the experiments were conducted three times (^*^P < 0.05, ^**^P < 0.01, ^***^P < 0.001, vs. 16HBE; ^#^*P* < 0.05, ^##^*P* < 0.01, ^###^*P* < 0.001, vs. BEAS2B).

We first determined the expression level of miR-486-3p in lung cancer on starBase (http://starbase.sysu.edu.cn/panMirDiffExp.php#). The results exhibited that miR-486-3p was low-expressed in both squamous cell carcinoma (*P* = 9.3e-5) and lung adenocarcinoma (*P* = 3.2e-5) as compared with normal tissues (Fig. 1A, B). QRT-PCR was performed to detect the miR-486-3p expression in the adjacent tissues and cancer tissues, and we found that miR-486-3p had a lower expression in the cancer tissues than in adjacent tissues (Fig. 1C). The expression level of miR-486-3p in a series of lung cancer cell lines (NCI-H520, SK-MES-1, H1299, A549, PC-9, and H23) and normal bronchial epithelial cell lines (16HBE and BEAS2B) (Fig. 1D) was determined, and it could found that miR-486-3p was also low-expressed in cancer cells as compared with normal bronchial epithelial cells. SK-MES-1 and A549 cells were therefore chosen for later use because the expression of miR-486-3p in the two cell lines was the lowest.

### MiR-486-3p mimic inhibited the viability, proliferation, migration, and invasion of SK-MES-1 and A549 cells

**Fig. 2:**
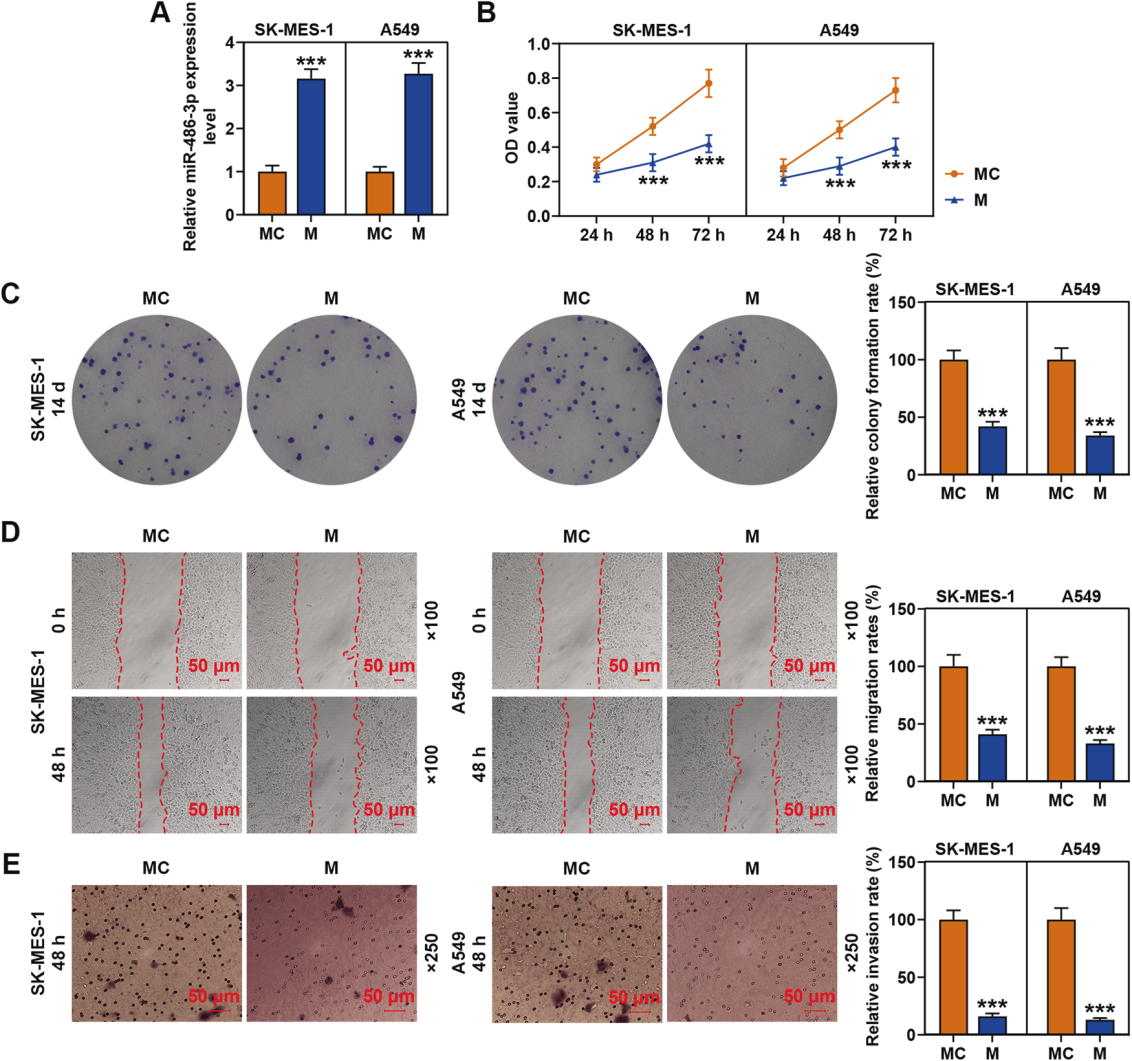
MiR-486-3p mimic inhibited the viability, proliferation, migration, and invasion of SK-MES-1 and A549 cells. **A** The transfection efficiency of miR-486-3p mimic in SK-MES-1 and A549 cells was evaluated by RT-qPCR. U6 was used as an internal control. **B** The viability of SK-MES-1 and A549 cells after transfection of miR-486-3p mimic was detected by CCK-8 assay. **C** The proliferation of SK-MES-1 and A549 cells after transfection of miR-486-3p mimic was detected by colony formation assay. **D** The migration of SK-MES-1 and A549 cells after transfection with miR-486-3p mimic was detected by wound-healing assay. **E** The invasion of SK-MES-1 and A549 cells after transfection of miR-486-3p mimic was detected by transwell assay. All the experiments were conducted three times (^***^P < 0.001, vs. MC). MC mimic control.

After the transfection of SK-MES-1 and A549 cells with miR-486-3p mimic, the transcription level of miR-486-3p was obviously increased as compared with the MC groups (Fig. 2A). Then we detected the effects of miR-486-3p on the viability of SK-MES-1 and A549 cells (Fig. 2B), and observed that miR-486-3p mimic significantly decreased the viabilities of the two cells after cell culture for 48 and 72 h as compared with the MC groups. Mechanically, the abilities of SK-MES-1 and A549 cells to proliferate, migrate, and invade after miR-486-3p mimic transfection (Fig. 2C–E) were also determined, and consistent with the results of cell viability detection, miR-486-3p mimic has been found to decrease the relative colony formation, migration, and invasion rates of the two cell lines. These results revealed that miR-486-3p mimic could inhibit the viability, proliferation, migration, and invasion of SK-MES-1 and A549 cells.

### CircFLNA sponged miR-486-3p in SK-MES-1 and A549 cells

**Fig. 3:**
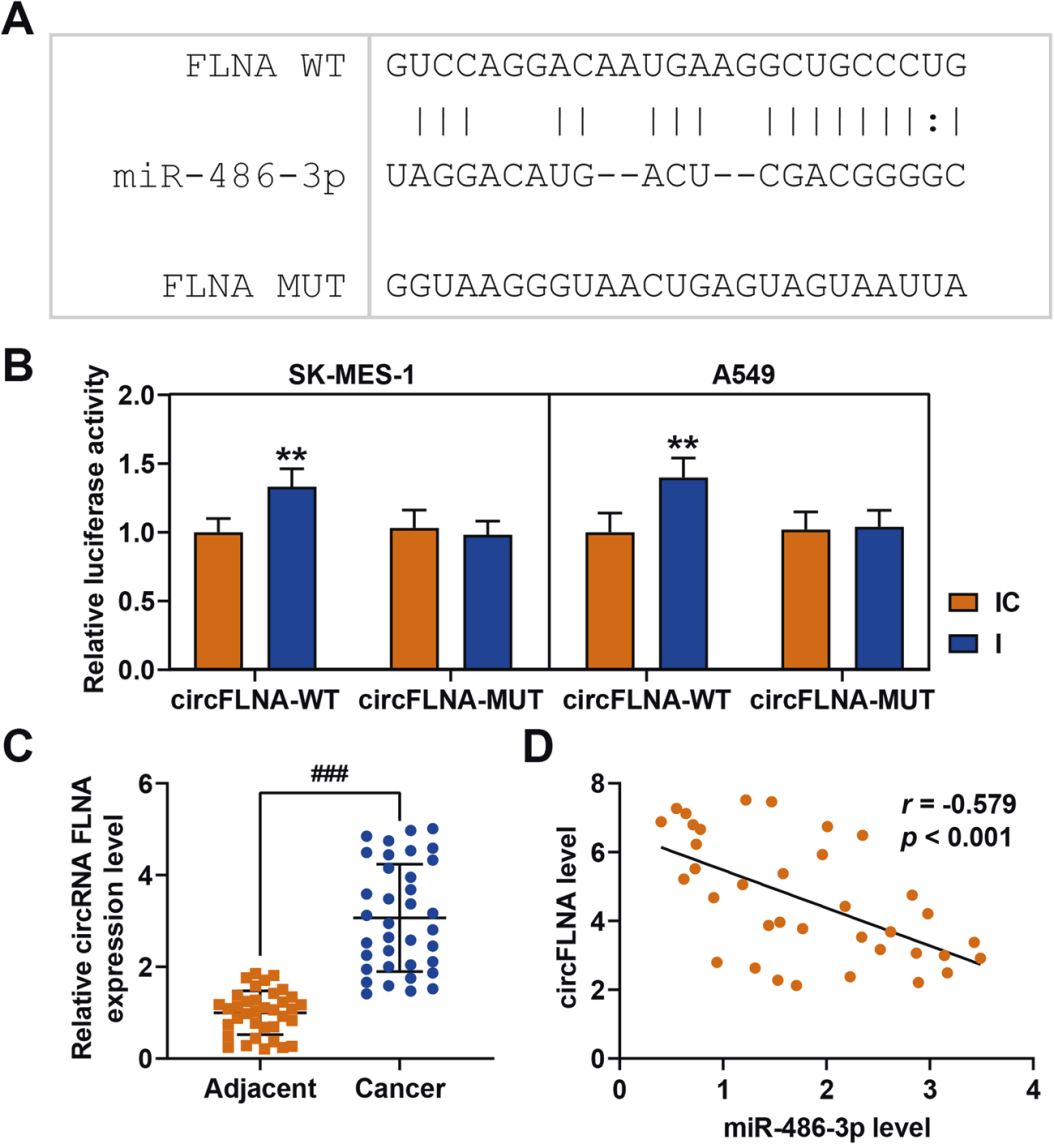
CircFLNA sponged miR-486-3p in SK-MES-1 and A549 cells. **A** The putative binding site between miR-486-3p and circFLNA was predicted by RNA22. **B** The result of luciferase reporter assay validated that circFLNA sponged miR-486-3p in SK-MES-1 and A549 cells. **C** QRT-PCR was used to detect the circFLNA expression in the cancer tissues and paracancer tissues. **D** Pearson was used to analyze the correlation between circFLNA and miR-486-3p. All the experiments were conducted three times (^**^P < 0.01, vs. IC). I miR-486-3p inhibitor, IC inhibitor control.

Considering that circRNAs regulate gene expression as miRNA sponges, we analyzed the potential binding circRNA for miR-486-3p. First, the circRNA binding sites in the miR-486-3p sequence was identified using two target prediction programs: RNA22 (https://cm.jefferson.edu/rna22/Interactive) and CircInteractome (https://circinteractome.nia.nih.gov/siRNA_Design/siRNA_design.html). As shown in Fig. 3A, miR-486-3p contained sequences complementary to circFLNA-WT. Luciferase reporter assay was performed to verify this prediction. As shown in Fig. 3B, luciferase activity was increased of both SK-MES-1 and A549 cells co-transfected with miR-486-3p inhibitor and circFLNA-WT as compared with that of cells co-transfected with inhibitor control and circFLNA-WT, while after transfection of miR-486-3p and circFLNA-MUT, there was no difference in the luciferase activity between SK-MES-1 and A549 cells co-transfected with miR-486-3p inhibitor and circFLNA-WT or those co-transfected with inhibitor control and circFLNA-WT. The data verified that circFLNA could sponge miR-486-3p in lung cancer cells. QRT-PCR was then used to detect the circFLNA expression in the cancer tissues and paracancer tissues, and the results showed that circFLNA expression was significantly increased in the cancer tissues (Fig. 3C). Moreover correlation analysis demonstrated that circFLNA was negatively correlated with miR-486-3p (Fig. 3D).

### CircFLNA was high-expressed in lung cancer cells and mainly located in the cytoplasm of SK-MES-1 and A549 cells

**Fig. 4:**
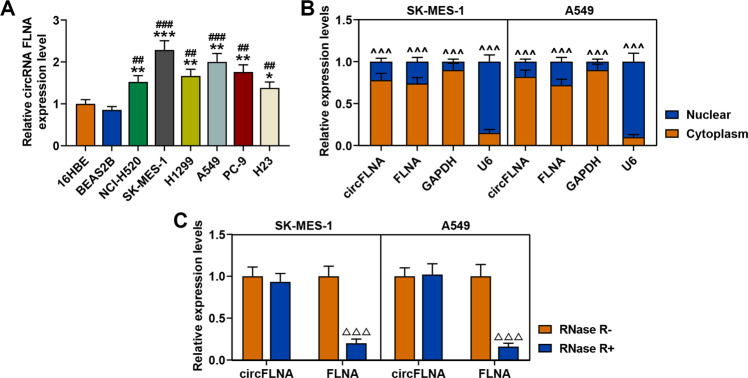
CircFLNA was high-expressed in lung cancer cells and mainly located in the cytoplasm of SK-MES-1 and A549 cells. **A** The expression level of circFLNA in lung cancer cell lines (NCI-H520, SK-MES-1, H1299, A549, PC-9, and H23) and normal bronchial epithelial cell lines (16HBE and BEAS2B) was determined by RT-qPCR. GAPDH was used as an internal control. All the experiments were conducted three times (^*^P < 0.05, ^**^P < 0.01, ^***^P < 0.001, vs. 16HBE; ^#^*P* < 0.05, ^##^*P* < 0.01, ^###^*P* < 0.001, vs. BEAS2B). **B** Cytoplasmic and nuclear RNA fractions were isolated from SK-MES-1 and A549 cells. Relative expression levels of circFLNA and FLNA in the cytoplasm or nucleus were examined by RT-qPCR. GAPDH was used as a cytoplasmic internal control, and U6 was used as a nuclear internal control (^^^^^*P* < 0.001, vs. cytoplasm). **C** The expressions of circFLNA and FLNA in SK-MES-1 and A549 cells treated with or without RNase R were detected by RT-qPCR. GAPDH was used as an internal control (^△△△^*P* < 0.001, vs. RNase R^−^). All experiments were conducted three times.

We then detected the expression level of circFLNA in a series of lung cancer cell lines and normal bronchial epithelial cell lines. As shown in Fig. 4A, circFLNA was also high-expressed in cancer cells as compared with normal bronchial epithelial cells. Considering that circFLNA was derived from the FLNA gene, the locations of circFLNA and FLNA in lung cancer cells was determined by conducting RT-qPCR to measure the expression level of circFLNA in the nuclear and cytoplasm of SK-MES1 and A549 cells. The results demonstrated that circFLNA and FLNA were both mainly located in the cytoplasm of the two cells (Fig. 4B). Next, we further found that after incubating the RNA with RNase R, the expression level of circFLNA showed no change, while the expression level of FLNA was significantly decreased in both SK-MES-1 and A549 cells as compared with the RNA without incubation with RNase R (Fig. 4C). These data confirmed that circFLNA was resistant to RNase R.

### CircFLNA knockdown increased the expression of miR-486-3p and inhibited the viability, proliferation, migration, and invasion of SK-MES-1 and A549 cells

**Fig. 5:**
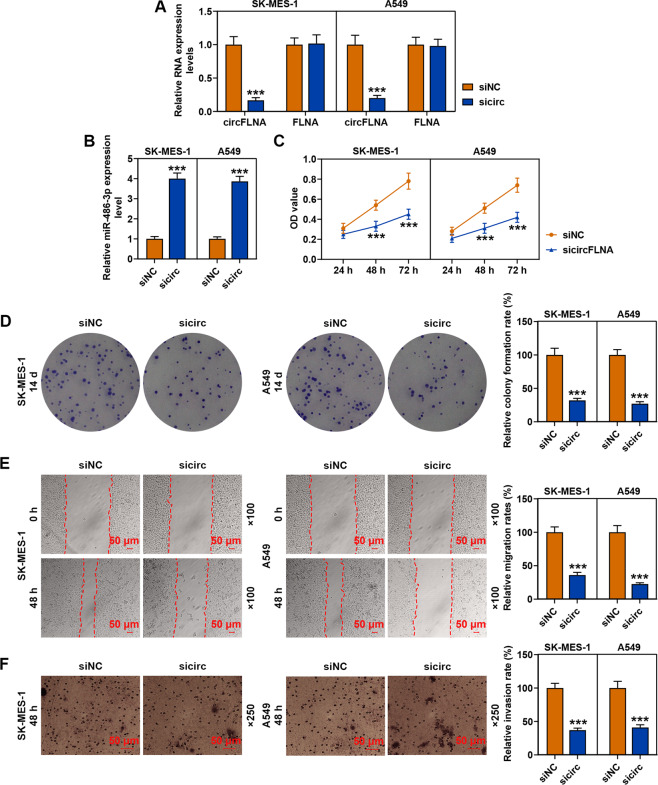
CircFLNA knockdown up-regulated the expression of miR-486-3p and inhibited the viability, proliferation, migration, and invasion of SK-MES-1 and A549 cells. **A** The transfection efficiency of sicircFLNA in SK-MES-1 and A549 cells was evaluated by RT-qPCR. GAPDH was used as an internal control. **B** The expression of miR-486-3p in SK-MES-1 and A549 cells after transfection of sicircFLNA was detected by RT-qPCR. U6 was used as an internal control. **C** The viability of SK-MES-1 and A549 cells after transfection of sicircFLNA was detected by CCK-8 assay. **D** The proliferation of SK-MES-1 and A549 cells after transfection of sicircFLNA was detected by colony formation assay. **E** The migration of SK-MES-1 and A549 cells after transfection with sicircFLNA was detected by wound-healing assay. **F** The invasion of SK-MES-1 and A549 cells after transfection of sicircFLNA was detected by transwell assay. All the experiments were conducted three times (^***^P < 0.001, vs. siNC). siNC small interfering RNA negative control.

After transfection of SK-MES-1 and A549 cells with sicircFLNA, the transcription level of circFLNA was obviously decreased as compared with the siNC groups, while the expression of FLNA showed no change (Fig. 5A), moreover, sicircFLNA also up-regulated the expression of miR-486-3p in both SK-MES-1 and A549 cells as compared with the siNC groups (Fig. 5B). Furthermore, we detected the effects of circFLNA on the viability of SK-MES-1 and A549 cells (Fig. 5C), and observed that sicircFLNA significantly decreased the viability of the two cells after cell culture for 48 and 72 h as compared with the siNC groups (*P* < 0.001). Mechanically, consistent with the resultd of cell viability detection, sicircFLNA decreased the relative colony formation, migration, and invasion rates of SK-MES-1 and A549 cells (Fig. 5D, F). All these findings revealed that sicircFLNA could promote the expression of miR-486-3p and inhibit the viability, proliferation, migration, and invasion of SK-MES-1 and A549 cells.

### MiR-486-3p mimic reversed the effect of circFLNA overexpression on inhibiting the expression of miR-486-3p and promoting the viability, proliferation, migration, and invasion of SK-MES-1 and A549 cells

**Fig. 6:**
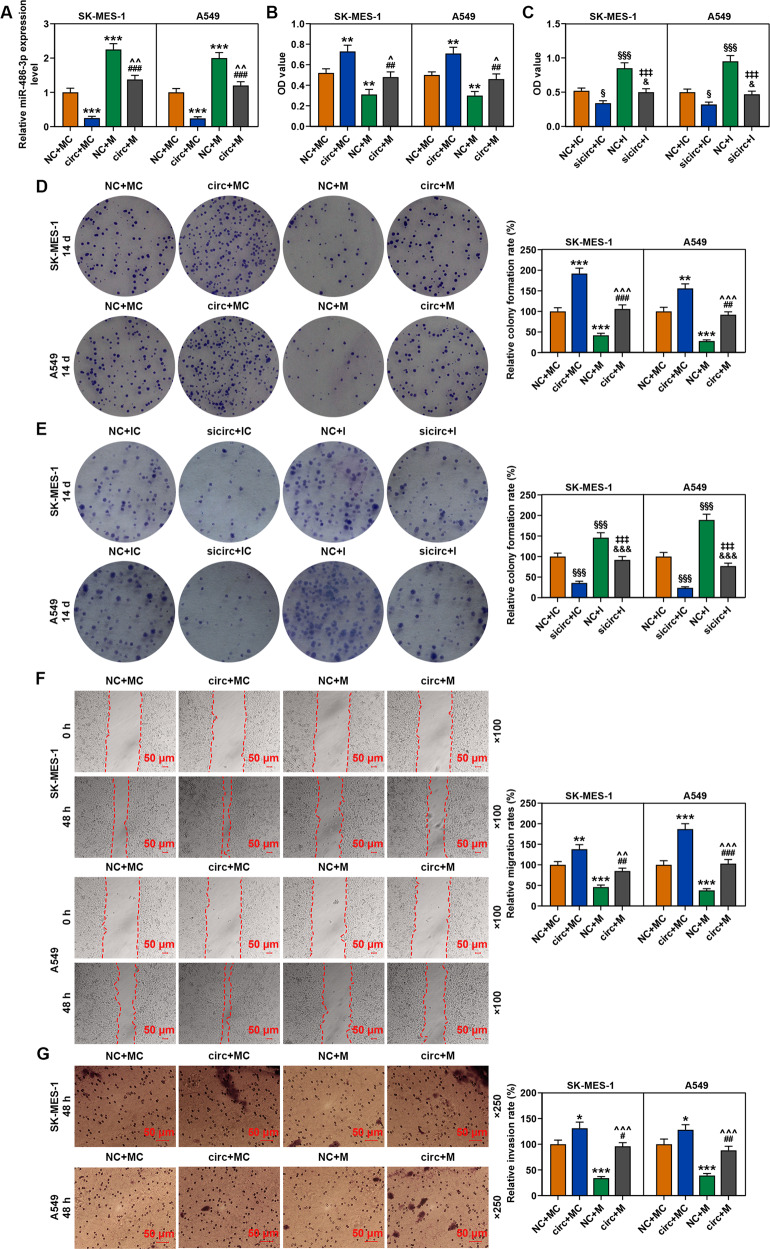
MiR-486-3p mimic reversed the effect of circFLNA overexpression on inhibiting the expression of miR-486-3p and promoting the viability, proliferation, migration, and invasion of SK-MES-1 and A549 cells. **A** The expression of miR-486-3p in SK-MES-1 and A549 cells after transfection was detected by RT-qPCR; U6 was used as an internal control. **B** The viability of SK-MES-1 and A549 cells after transfection was detected by CCK-8 assay. **C** CCK-8 was used to detect the viability of SK-MES-1 and A549 cells transfected with circFLNA and inhibitor. **D** The proliferation of SK-MES-1 and A549 cells after transfection was detected by colony formation assay. **E** The colony formation assay was used to detect the proliferation of SK-MES-1 and A549 cells transfected with circFLNA and inhibitor. **F** The migration of SK-MES-1 and A549 cells after transfection was detected by wound-healing assay. **G** The invasion of SK-MES-1 and A549 cells after transfection was detected by transwell assay. All the experiments were conducted three times (^*^P < 0.05, ^**^P < 0.01, ^***^P < 0.001, vs. NC + MC; ^#^*P* < 0.05, ^##^*P* < 0.01, ^###^*P* < 0.001, vs. circ+MC; ^^^*P* < 0.05, ^^^^*P* < 0.01, ^^^^^*P* < 0.001, vs. NC + M). circ circFLNA, NC overexpression negative control, circ circFLNA, M miR-486-3p mimic, MC mimic control.

We then co-transfected circFLNA with miR-486-3p mimic into SK-MES-1 and A549 cells to investigate the correlation between circFLNA and miR-486-3p. As shown in Fig. 6A, the expression of miR-486-3p was decreased by circFLNA overexpression but increased by miR-486-3p mimic as compared with the NC + MC groups, while after the co-transfection of circFLNA and miR-486-3p mimic (circ + M group), the inhibitory effect of circFLNA overexpression on miR-486-3p expression in both SK-MES-1 and A549 cells was reversed by miR-486-3p mimic as compared with the circ+MC and NC + M groups. As shown in Fig. 6B, the viability of the two cells was increased by circFLNA overexpression but decreased by miR-486-3p mimic as compared with the NC + MC groups, while after co-transfection of circFLNA and miR-486-3p mimic (circ + M group), the promoting effect of circFLNA overexpression on cell viability of both SK-MES-1 and A549 cells was reversed by miR-486-3p mimic as compared with the circ+MC and NC + M groups. Overexpression of circFLNA promoted cell viability, and the inhibitor further enhanced the effect of circFLNA on cell viability (Fig. 6C). Consistent with the result of cell viability, the relative colony formation (Fig. 6D, E), migration (Fig. 6F), and invasion rates (Fig. 6G) of the two cells were promoted by circFLNA overexpression and inhibited by miR-486-3p mimic as compared with the NC + MC groups, while after co-transfection of circFLNA and miR-486-3p mimic (circ + M group), the promoting effect of circFLNA on both SK-MES-1 and A549 cells was reversed by miR-486-3p mimic as compared with the circ+MC and NC + M groups. Also, overexpression of circFLNA promoted cell colony formation, and the inhibitor further enhanced the effect of circFLNA on cell colony formation (Fig. 6E).

### MiR-486-3p mimic reversed the effect of circFLNA overexpression on inhibiting the expression of E-Cadherin and promoting the expressions of N-Cadherin and Vimentin in SK-MES-1 and A549 cells

**Fig. 7:**
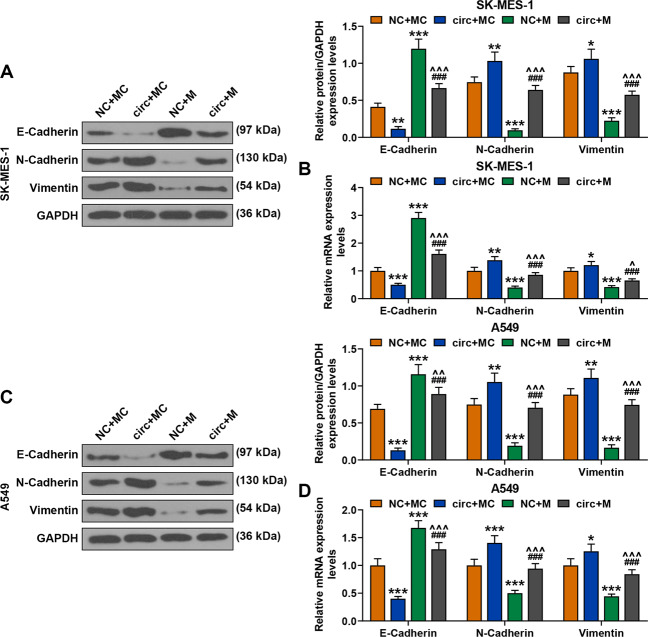
MiR-486-3p mimic reversed the effect of circFLNA overexpression on inhibiting the expression of E-Cadherin and promoting the expressions of N-Cadherin and Vimentin in SK-MES-1 and A549 cells. **A** The expressions of E-Cadherin, N-Cadherin, and Vimentin in SK-MES-1 cells after transfection were detected by western blot. GAPDH was used as an internal control. **B** The expressions of E-Cadherin, N-Cadherin, and Vimentin in SK-MES-1 cells after transfection were detected by RT-qPCR. GAPDH was used as an internal control. **C** The expressions of E-Cadherin, N-Cadherin, and Vimentin in A549 cells after transfection were detected by western blot. GAPDH was used as an internal control. **D** The expressions of E-Cadherin, N-Cadherin, and Vimentin in A549 cells after transfection were detected by RT-qPCR. GAPDH was used as an internal control. All the experiments were conducted three times (^*^P < 0.05, ^**^P < 0.01, ^***^P < 0.001, vs. NC + MC; ^###^*P* < 0.001, vs. circ+MC; ^^^*P* < 0.05, ^^^^*P* < 0.01, ^^^^^*P* < 0.001, vs. NC + M). circ circFLNA, NC overexpression negative control, circ circFLNA, M miR-486-3p mimic, MC mimic control.

We also detected the expressions of related factors in SK-MES-1 and A549 cells after co-transfection of circFLNA and miR-486-3p mimic. As shown in Fig. 7, the protein and gene expressions of E-Cadherin were decreased by circFLNA overexpression but increased by miR-486-3p mimic as compared with the NC + MC groups, while after co-transfection of circFLNA and miR-486-3p mimic (circ + M group), the inhibitory effect of circFLNA overexpression on E-Cadherin expressions in both SK-MES-1 and A549 cells was reversed by miR-486-3p mimic as compared with the circ+MC and NC + M groups. Meanwhile, the protein and gene expressions of N-Cadherin and Vimentin were increased by circFLNA overexpression and decreased by miR-486-3p mimic as compared with the NC + MC groups, while after co-transfection of circFLNA and miR-486-3p mimic (circ + M group), the inhibitory effect of overexpressed circFLNA on the expressions of N-Cadherin and Vimentin in both SK-MES-1 and A549 cells was reversed by miR-486-3p mimic as compared with the circ+MC and NC + M groups.

### MiR-486-3p specifically targeted XRCC1 and CYP1A1 in SK-MES-1 and A549 cells

**Fig. 8:**
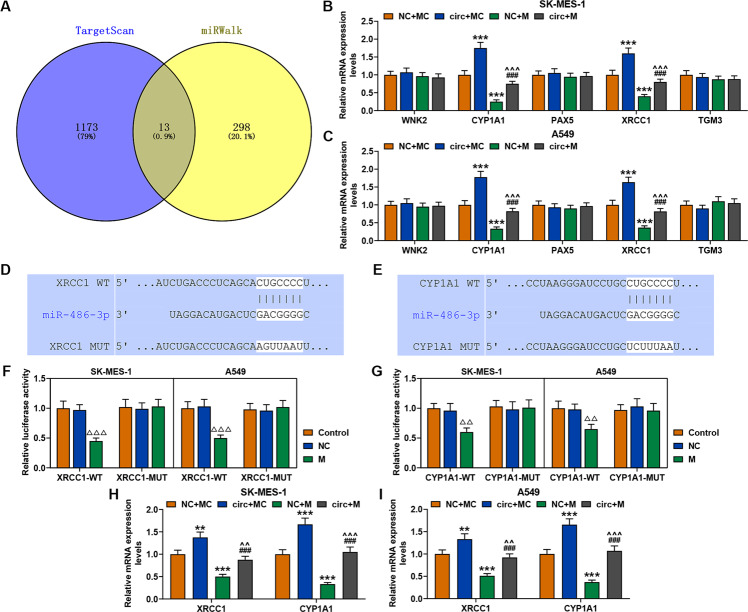
MiR-486-3p specifically targeted XRCC1 and CYP1A1 in SK-MES-1 and A549 cells. **A** The target genes of miR-486-3p were predicted using targetScan and miRWalk. **B**, **C** The expressions of WNK2, CYP1A1, PAX5, XRCC1, and TGM3 in SK-MES-1 cells after transfection were detected by western blot. GAPDH was used as an internal control (^***^P < 0.001, vs. NC + MC; ^###^*P* < 0.001, vs. circ+MC; ^^^^^*P* < 0.001, vs. NC + M). **D**, **E** The putative binding site between miR-486-3p and XRCC1 or XRCC1 was predicted using targetScan. **F**, **G** The result of Luciferase reporter assay validated that miR-486-3p specifically targeted XRCC1 and CYP1A1 in SK-MES-1 and A549 cells (^△△△^*P* < 0.001, ^△△^*P* < 0.01, vs. NC). **H** The expressions of XRCC1 and CYP1A1 in the SK-MES-1 cells transfected with circFLNA and mimic were detected by qRT-PCR. **I** The expression of XRCC1 and CYP1A1 in the A549 cells transfected with circFLNA and mimic were detected by qRT-PCR. circ circFLNA, NC overexpression negative control, circ circFLNA, M miR-486-3p mimic, MC mimic control.

The targets of miR-486-3p were predicted through targetScan and miRWalk, and the results predicted 13 common target genes (Fig. 8A). Considering that the expression levels of WNK2, CYP1A1, PAX5, XRCC1, and TGM3 have been reported to be closely related to the development of cancer [16–19], we then detected the expression levels of these genes in SK-MES-1 and A549 cells after transfection of circFLNA and miR-486-3p mimic. It has been observed that only the expressions of XRCC1 and CYP1A1 could be regulated by circFLNA and miR-486-3p mimic in both SK-MES-1 and A549 cells (Fig. 8B, C). Specifically, the expressions of XRCC1 and CYP1A1 were up-regulated by circFLNA overexpression and down-regulated by miR-486-3p mimic, while after co-transfection of circFLNA and miR-486-3p mimic, the promoting effect of circFLNA overexpression was reversed by miR-486-3p mimic (Fig. 8B, C). In addition, the prediction of targetScan exhibited that XRCC1 and CYP1A1 were the targets of miR-486-3p because miR-486-3p contained sequences complementary to XRCC1-WT and CYP1A1-WT (Fig. 8D, E). To verify this prediction, we conducted luciferase reporter assay, and found that luciferase activity was decreased in both SK-MES-1 and A549 cells co-transfected with miR-486-3p mimic and XRCC1-WT (Fig. 8F) or CYP1A1-WT (Fig. 8G), while after co-transfection of miR-486-3p mimic and XRCC1-MUT (Fig. 8F) or CYP1A1-MUT (Fig. 8G), there was no difference in the luciferase activity. These findings further verified that miR-486-3p specifically targeted XRCC1 and CYP1A1 in SK-MES-1 and A549 cells. Moreover, the expressions of XRCC1 and CYP1A1 in cells transfected with circFLNA and mimic were detected by qRT-PCR, and the data showed that overexpression of circFLNA promoted the expressions of XRCC1 and CYP1A1, while mimic led to opposite results (Fig. 8H, I).

### Overexpressed XRCC1 and CYP1A1 reversed the inhibitory effect of miR-486-3p mimic on the viability, proliferation, migration, and invasion of SK-MES-1 and A549 cells

**Fig. 9:**
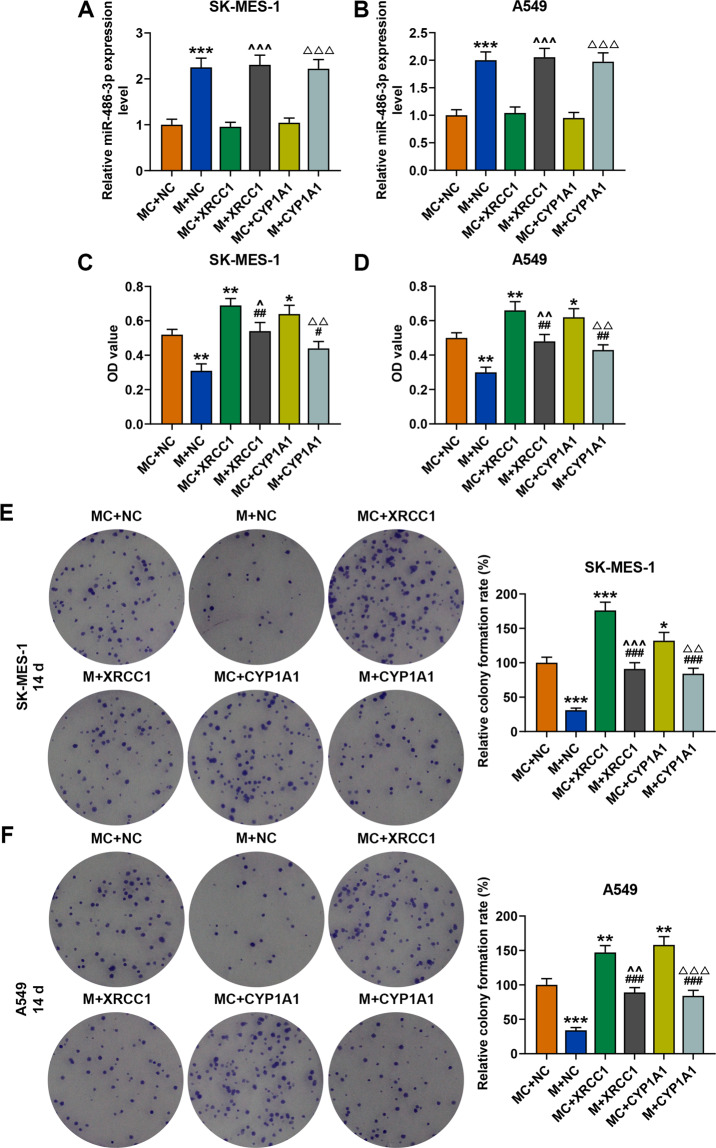
Overexpressions of XRCC1 and CYP1A1 reversed the inhibitory effect of miR-486-3p mimic on the viability and proliferation of SK-MES-1 and A549 cells. **A**, **B** The expression of miR-486-3p in SK-MES-1 and A549 cells after transfection was detected by RT-qPCR. U6 was used as an internal control. **C**, **D** The viability of SK-MES-1 and A549 cells after transfection was detected by CCK-8 assay. **E**, **F** The proliferation of SK-MES-1 and A549 cells after transfection was detected by colony formation assay. All the experiments were conducted three times (^*^P < 0.05, ^**^P < 0.01, ^***^P < 0.001, vs. MC + NC; ^#^*P* < 0.05, ^##^*P* < 0.01, ^###^*P* < 0.001, vs. M + NC; ^^^*P* < 0.05, ^^^^*P* < 0.01, ^^^^^*P* < 0.001, vs. MC + XRCC1; ^△^*P* < 0.05, ^△△^*P* < 0.01, ^△△△^*P* < 0.001, vs. MC + CYP1A1). NC overexpression negative control, M miR-486-3p mimic, MC mimic control.

**Fig. 10:**
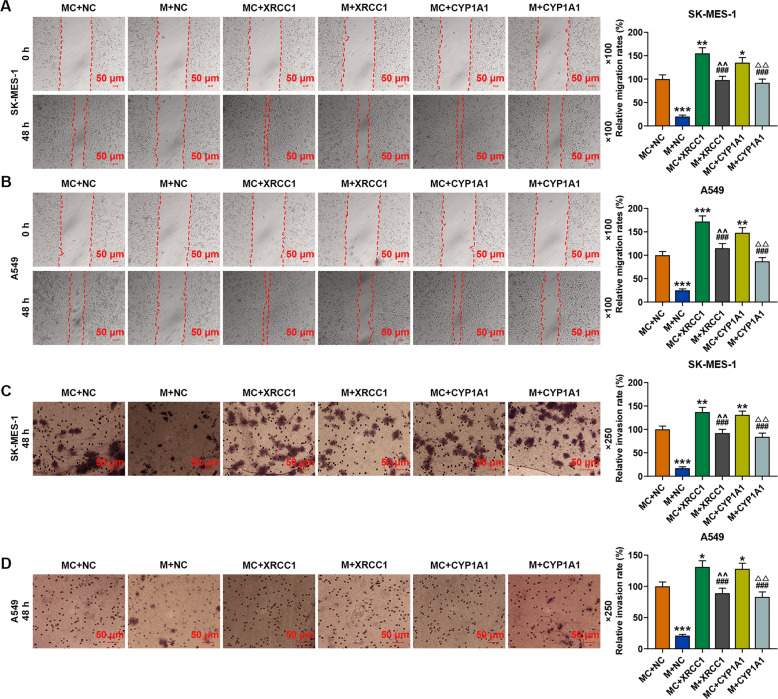
Overexpression of XRCC1 and CYP1A1 reversed the inhibitory effect of miR-486-3p mimic on the migration and invasion of SK-MES-1 and A549 cells. **A**, **B** The migration of SK-MES-1 and A549 cells after transfection was detected by wound-healing assay. **C**, **D** The invasion of SK-MES-1 and A549 cells after transfection was detected by transwell assay. All the experiments were conducted three times (^*^P < 0.05, ^**^P < 0.01, ^***^P < 0.001, vs. MC + NC; ^###^*P* < 0.001, vs. M + NC; ^^^^*P* < 0.01, vs. MC + XRCC1; ^△△^*P* < 0.01, vs. MC + CYP1A1). NC overexpression negative control, M miR-486-3p mimic, MC mimic control.

As shown in Fig. 9A, B, overexpressed XRCC1 and CYP1A1 did not affect the expression of miR-486-3p in SK-MES-1 or A549 cells. We then detected the effects of XRCC1 and CYP1A1 on the viability, proliferation, migration, and invasion of SK-MES-1 and A549 cells. As shown in Fig. 9C, D, the viability of the two cells was inhibited by miR-486-3p mimic but enhanced by overexpressed XRCC1 and CYP1A1 as compared with the NC + MC groups, while after co-transfection of miR-486-3p mimic and XRCC1 (M + XRCC1 group) or CYP1A1 (M + CYP1A1 group), the inhibitory effect of miR-486-3p mimic on the viability of both SK-MES-1 and A549 cells was reversed by overexpressed XRCC1 and CYP1A1. Similar to the result of cell viability detection, the relative colony formation (Fig. 9E, F), migration (Fig. 10A, B), and invasion (Fig. 10C, D) rates of the two cells were decreased by miR-486-3p mimic but promoted by overexpressions of XRCC1 and CYP1A1, while after co-transfection of miR-486-3p mimic and XRCC1 or CYP1A1, the inhibitory effect of miR-486-3p mimic was reversed by overexpression of XRCC1 and CYP1A1 in both SK-MES-1 and A549 cells.

### Overexpressions of XRCC1 and CYP1A1 reversed the effect of miR-486-3p mimic on promoting the expression of E-Cadherin and inhibiting the expressions of N-Cadherin and Vimentin in SK-MES-1 and A549 cells

**Fig. 11:**
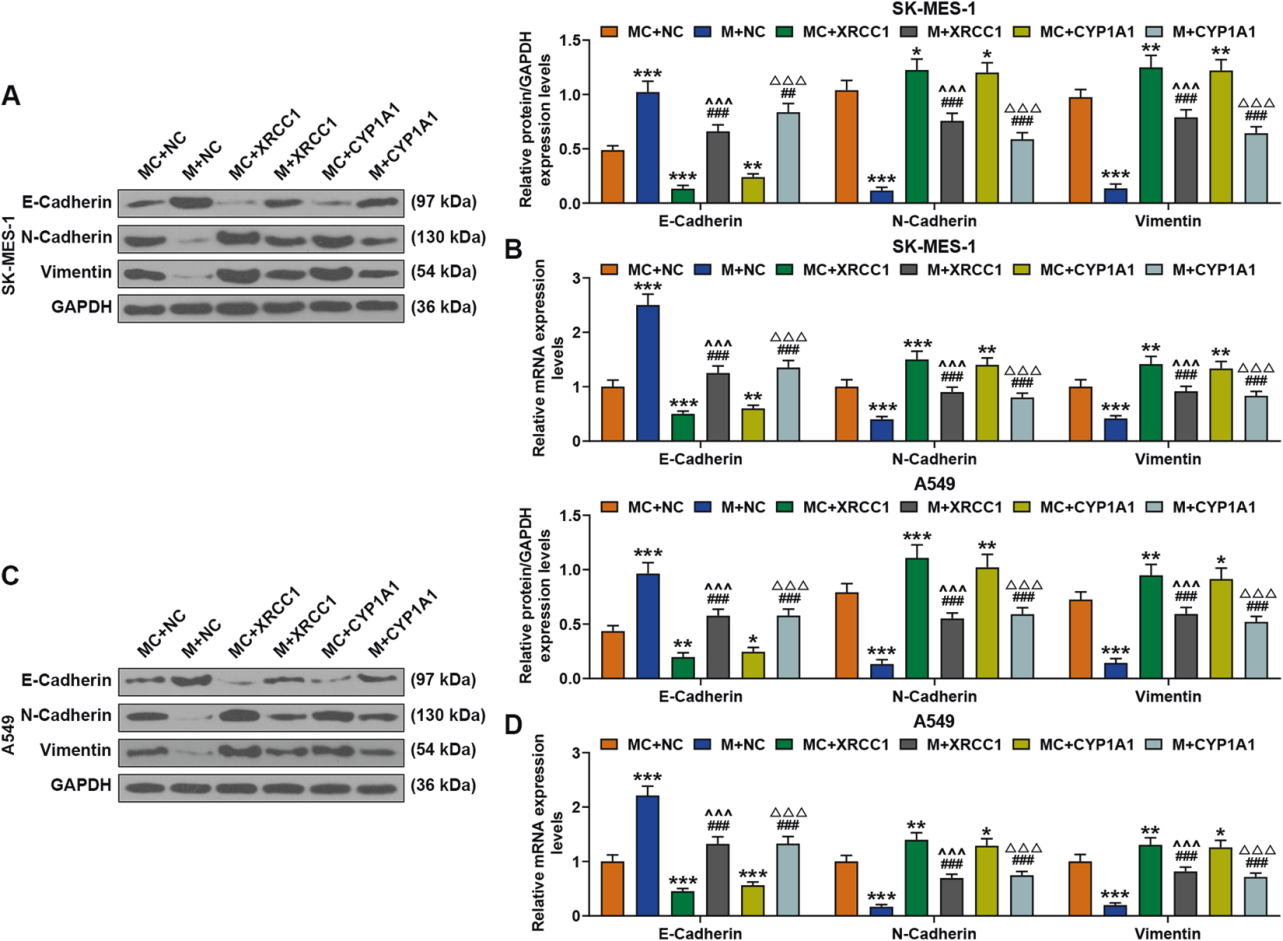
Overexpression of XRCC1 and CYP1A1 reversed the effect of miR-486-3p mimic on promoting the expression of E-Cadherin and inhibiting the expressions of N-Cadherin and Vimentin in SK-MES-1 and A549 cells. **A** The expressions of E-Cadherin, N-Cadherin, and Vimentin in SK-MES-1 cells after transfection were detected by Western blot. GAPDH was used as an internal control. **B** The expressions of E-Cadherin, N-Cadherin, and Vimentin in SK-MES-1 cells after transfection were detected by RT-qPCR. GAPDH was used as an internal control. **C** The expressions of E-Cadherin, N-Cadherin, and Vimentin in A549 cells after transfection were detected by Western blot. GAPDH was used as an internal control. **D** The expressions of E-Cadherin, N-Cadherin, and Vimentin in A549 cells after transfection were detected by RT-qPCR. GAPDH was used as an internal control. All the experiments were conducted three times (^*^P < 0.05, ^**^P < 0.01, ^***^P < 0.001, vs. MC + NC; ^##^*P* < 0.01,^###^*P* < 0.001, vs. M + NC; ^^ ^^^*P* < 0.001, vs. MC + XRCC1; ^△△△^*P* < 0.001, vs. MC + CYP1A1). NC overexpression negative control, M miR-486-3p mimic, MC mimic control.

As shown in Fig. 11, the protein and gene expressions of E-Cadherin were increased by miR-486-3p mimic but decreased by overexpressions of XRCC1 and CYP1A1, while after co-transfection of miR-486-3p mimic, XRCC1 and CYP1A1, the promoting effect of miR-486-3p mimic on E-Cadherin expressions in both SK-MES-1 and A549 cells was reversed by overexpressed XRCC1 and CYP1A1. The protein and gene expressions of N-Cadherin and Vimentin were decreased by miR-486-3p mimic but increased by overexpressed XRCC1 and CYP1A1, while after co-transfection of miR-486-3p mimic, XRCC1 and CYP1A1, the inhibitory effect of miR-486-3p mimic on the expressions of N-Cadherin and Vimentin in both SK-MES-1 and A549 cells was reversed by overexpressed XRCC1 and CYP1A1.

### MiR-486-3p mimic reversed the effect of overexpressed circFLNA on promoting tumor growth and regulating the expressions of apoptosis-related factors

**Fig. 12:**
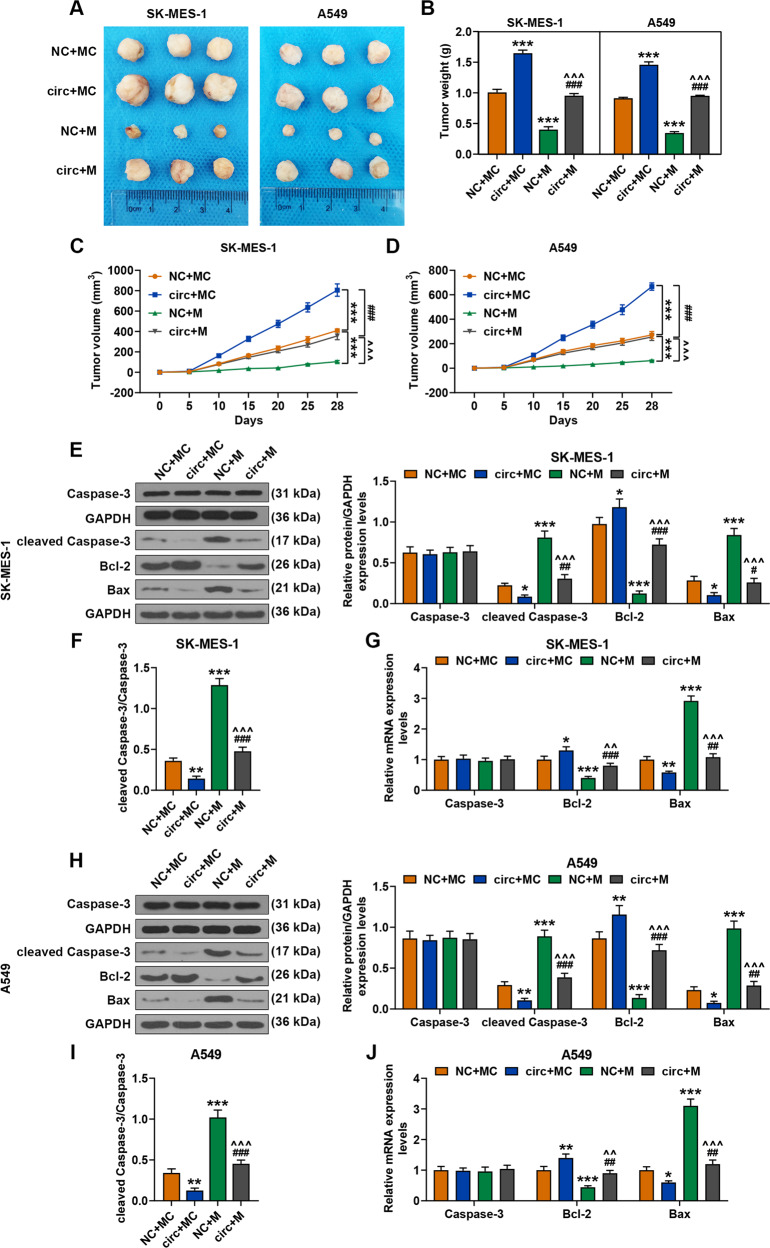
MiR-486-3p mimic reversed the effect of circFLNA on promoting tumor growth and regulating the expression of apoptosis factors. **A** The picture of solid tumor was exhibited and the tumor weight was calculated. **B** The tumor weight of subcutaneous xenograft mouse model was calculated. **C**, **D** The tumor volume of subcutaneous xenograft mouse model was calculated at day 5, 10, 15, 20, 25, and 28, respectively. **E** The expressions of cleaved Caspase-3, caspase 3, Bcl-2, and Bax in the tissues from mice injected with SK-MES-1 cells were detected by Western blot. GAPDH was used as an internal control. **F** The level of cleaved caspase-3/caspase-3 was analyzed quantitatively. **G** The expressions of cleaved Caspase-3, caspase 3, Bcl-2, and Bax in tissue from mice injected with SK-MES-1 cells were detected by RT-qPCR. GAPDH was used as an internal control. **H** The expressions of cleaved Caspase-3, caspase 3, Bcl-2, and Bax in the tissues from mice injected with A549 cells were detected by western blot. GAPDH was used as an internal control. **I** The level of cleaved caspase-3/caspase-3 was analyzed quantitatively in the tissues from mice injected with A549 cells. **J** The expressions of cleaved Caspase-3, caspase 3, Bcl-2, and Bax in the tissues from mice injected with A549 cells were detected by RT-qPCR. GAPDH was used as an internal control. (^*^P < 0.05, ^**^P < 0.01, ^***^P < 0.001, vs. NC + MC; ^#^*P* < 0.05, ^##^*P* < 0.01, ^###^*P* < 0.001, vs. circ+MC; ^^^^*P* < 0.01, ^^^^^*P* < 0.001, vs. NC + M). circ circFLNA overexpression, NC overexpression negative control, M miR-486-3p mimic, MC mimic control.

As exhibited in Fig. 12A, B, the tumor weight was increased by circFLNA overexpression but decreased by miR-486-3p mimic as compared with the NC + MC groups, while after co-transfection of miR-486-3p mimic and circFLNA, the promoting effect of circFLNA overexpression on tumor growth was reversed by miR-486-3p mimic. From day 5 to 28, overexpressed circFLNA significantly promoted tumor formation, while the overexpressed Mir-486-3p greatly reversed the promotion resulted from circFLNA (*P* < 0.001, Fig. 12C, D). As shown in Fig. 12E, G, the expressions of cleaved Caspase-3 and Bax in tissues derived from the mice injected with SK-MES-1 cells were reduced by circFLNA overexpression and increased by miR-486-3p mimic as compared with the NC + MC groups, while after co-transfection of miR-486-3p mimic and circFLNA, the inhibitory effect of circFLNA overexpression on the expressions of cleaved Caspase-3 and Bax was reversed by miR-486-3p mimic. Western blot was performed to detect the expression of caspase 3 in the tissues derived from mice injected with SK-MES-1 cells, and the results showed that the ratio of cleaved caspase-3/caspase-3 was significantly increased in the mimic group, while overexpression of circFLNA could reverse the effect of mimic on the activation of cleaved caspase-3 in the tissues from mice injected with SK-MES-1 cells (Fig. 12F). In addition, the expression of Bcl-2 among these groups showed an opposite tendency to that of Bax expression (Fig. 12E, G). Similarly, the expression of cleaved Caspase-3, Bcl-2 and Bax, and the ratio of cleaved caspase-3/caspase-3 were consistent with those detected in the tissues from mice injected with A549 cells (Fig. 12H–J).

### MiR-486-3p mimic reversed the regulatory effect of circFLNA on the expressions of circFLNA, miR-486-3p, XRCC1, CYP1A1, Ki67, and PCNA in the tumor tissues

**Fig. 13:**
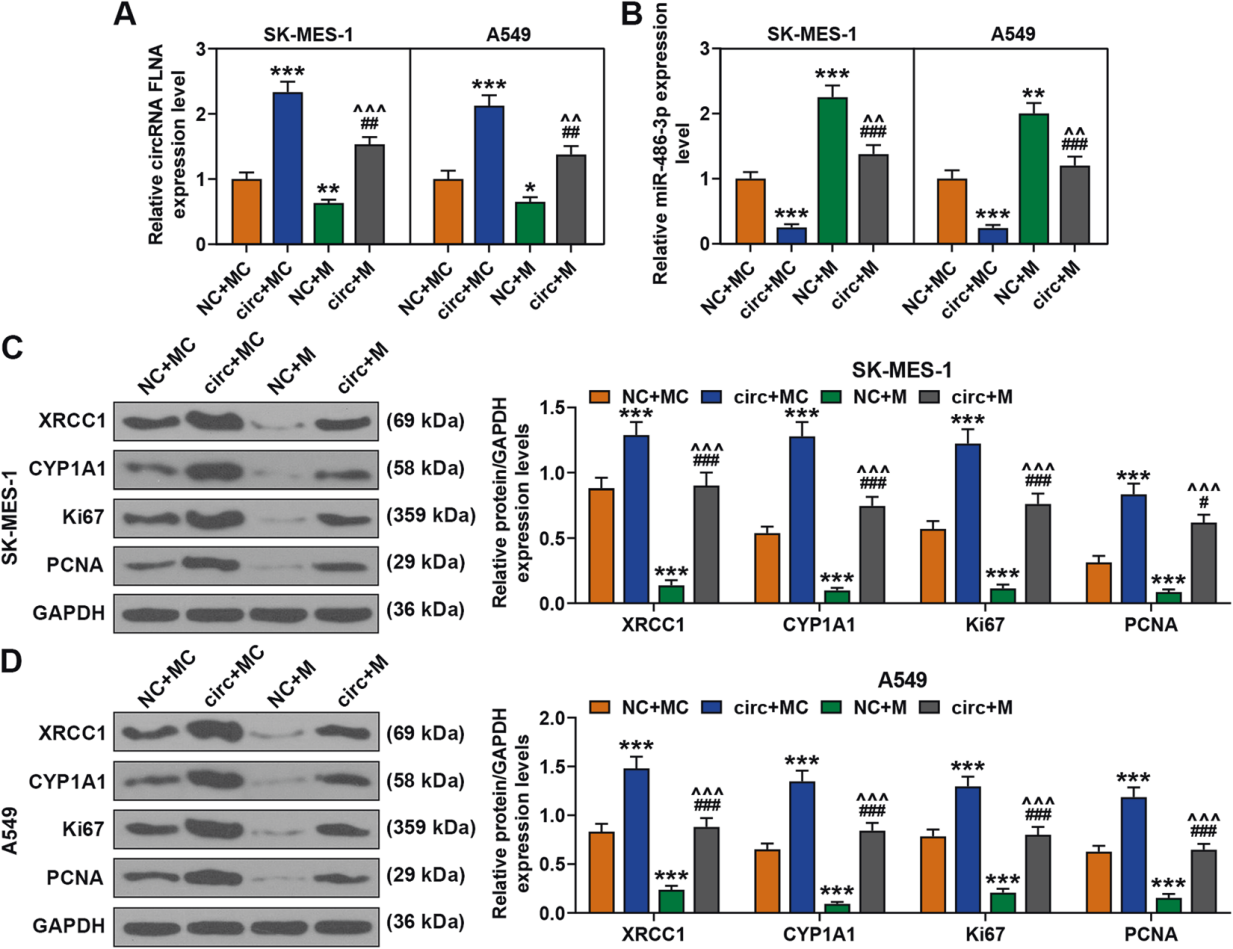
MiR-486-3p mimic reversed the regulatory effect of circFLNA on the expression of circFLNA, miR-486-3p, XRCC1, CYP1A1, Ki67, and PCNA in tumor tissues. **A** The expressions of circFLNA in tumor tissues were detected by RT-qPCR. GAPDH was used as an internal control. **B** The expressions of miR-486-3p in tumor tissues were detected by RT-qPCR. U6 was used as an internal control. **C**, **D** The expressions of XRCC1, CYP1A1, Ki67, and PCNA in tumor tissues were detected by western blot. GAPDH was used as an internal control. immunohistochemistry. (^*^P < 0.05, ^**^P < 0.01, ^***^P < 0.001, vs. NC + MC; ^#^*P* < 0.05, ^##^*P* < 0.01, ^###^*P* < 0.001, vs. circ+MC; ^^^^*P* < 0.01, ^^^^^*P* < 0.001, vs. NC + M). circ circFLNA overexpression, NC overexpression negative control, M miR-486-3p mimic, MC mimic control.

**Fig. 14:**
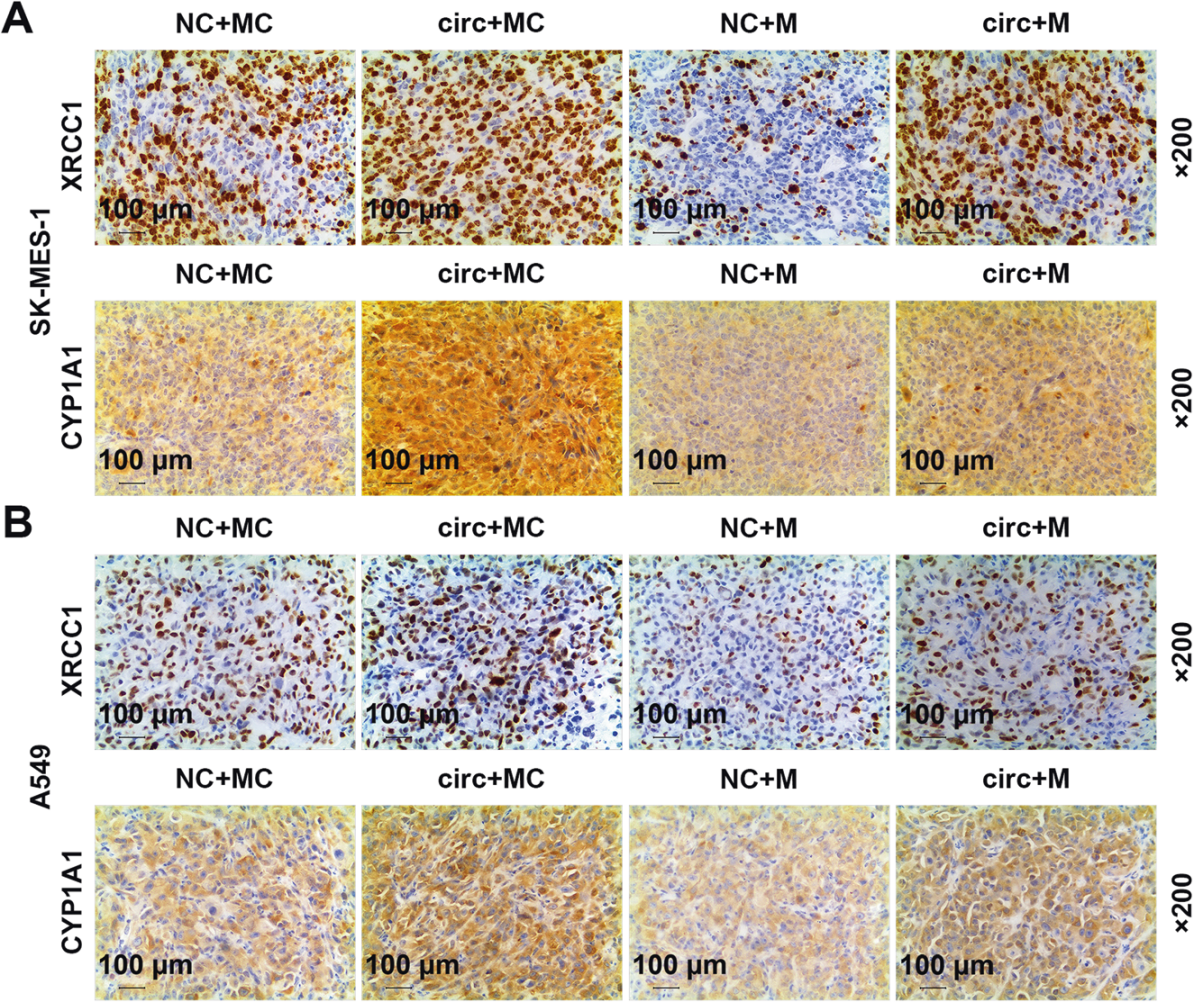
MiR-486-3p mimic reversed the regulatory effect of circFLNA on the expressions of Ki67 and PCNA in tumor tissues. **A**, **B** The expressions of Ki67 and PCNA in tumor tissues were detected by immunohistochemistry. circ circFLNA overexpression, NC overexpression negative control, M miR-486-3p mimic, MC mimic control.

As exhibited in Fig. 13A, B, circFLNA overexpression increased the expression of circFLNA and reduced the expression of miR-486-3p, but miR-486-3p mimic reduced the expression of circFLNA and increased the expression of miR-486-3p. After co-transfection of miR-486-3p mimic and circFLNA, the regulatory effect of circFLNA overexpression on the expressions of circFLNA and miR-486-3p was reversed by miR-486-3p mimic. In addition, as shown in Fig. 13C, D, the expressions of XRCC1, CYP1A1, Ki67, and PCNA were all increased by circFLNA overexpression and reduced by miR-486-3p mimic, while after co-transfection of miR-486-3p mimic and circFLNA, the promoting effect of circFLNA overexpression on the expressions of these factors was reversed by miR-486-3p mimic. Also the expressions of Ki-67 and PCNA in tumor tissues were further verified by performing immunohistochemistry (Fig. 14).

## Discussion

Increasing evidence has proven that abnormal expressions of miRNAs are highly related to the occurrence and development of various diseases and cancers, including lung cancer [9, 10, 20–22]. In this study, we first found that miR-486-3p was low-expressed in lung cancer tissues and cells. MiR-486-3p has been previously reported abnormally expressed in various diseases, including in cervical cancer, laryngeal carcinoma, and oral cancer [23–25]. In addition, microarray analysis also predicted low-expressed miR-486-3p in lung cancer [13]. Our study revealed for the first time that the abnormal expression of miR-486-3p was involved in lung cancer. However, the effect of miR-486-3p on lung cancer requires more exploration. The difficulties in the treatment of lung cancer are mostly resulted from rapidly proliferating lung cancer cells and their infiltration into normal tissues [26]. Thus, we focused on detecting the effect of miR-486-3p on the proliferation, migration, and invasion of lung cancer cells, and the results demonstrated that miR-486-3p mimic could inhibit proliferation, migration and invasion of lung cancer cells. These findings further encouraged us to investigate the underlying effect mechanisms through which miR-486-3p had a regulatory effect on lung cancer cells.

CircRNAs, which are a conserved and stable type of endogenous non-coding RNAs, are formed by back-splicing events of precursor mRNA. CircRNAs play important roles in the carcinogenesis and development of many cancers [27]. Among the researched circRNAs, only circFLNA was reported to be abnormally expressed in human oral and laryngeal squamous cell carcinomas [28–30]. In this study, for the first time, our group found that abnormally up-regulated circFLNA was also related to lung cancer. In recent years, the role of “miRNA sponges” has been frequently discussed as some circRNAs have been found to possess miRNA binding sites [5]. In the current study, bioinformatics was applied to determine whether circFLNA could act as a sponge to affect the expression of miR-486-3p in lung cancer, and it was predicted that the linear sequence of circFLNA shared binding sites with miR-486-3p, which was further verified by luciferase reporter assay. In addition, circFLNA significantly decreased the expression of miR-486-3p, confirming that circFLNA was a sponge of miR-486-3p, and suggesting that circFLNA might play a crucial role in the biological functions of lung cancer cells. Previous study reported that circFLNA could enhance the migration of human laryngeal squamous cell carcinoma cells [29]. Similar to the above research, this study showed that circFLNA overexpression promoted the viability, proliferation, migration, and invasion of lung cancer cells by regulating related genes, while circFLNA knockdown had an opposite effect on lung cancer cells. In addition, miR-486-3p mimic reversed the promoting effect of circFLNA overexpression on lung cancer cells, further proving that circFLNA acted as a sponge of miR-486-3p to regulate the proliferation, migration, and invasion of lung cancer cells. However, the underlying mechanism through which circFLNA/miR-486-3p signal affected lung cancer still needs further investigation.

Mounting evidence has confirmed that miRNAs could target certain mRNAs and further regulate the development of serious diseases [31–33]. To further clarify the effect of miR-486-3p on lung cancer cells, bioinformatics and luciferase reporter assays were applied. The data revealed that XRCC1 and CYP1A1 were the targets of miR-486-3p in lung cancer cells. XRCC1 is an important factor participating in repairing DNA damage and contributes to chemoresistance in serious cancers such as liver and lung cancers [34, 35]. CYP1A1 is an important phase I enzyme of the cytochrome P450 superfamily, and plays a key role in the detoxification of several xenobiotics and endogenous substances [36]. Here, we further demonstrated that overexpressions of XRCC1 and CYP1A1 enhanced the viability, proliferation, migration, and invasion of lung cancer cells through regulating related genes. In addition, overexpressed of XRCC1 and CYP1A1 reversed the inhibitory effect of miR-486-3p mimic on lung cancer cells, which further confirmed the targeted relationship among miR-486-3p and XRCC1 and CYP1A1.

To verify the results in this study in vivo, we also established a nude mice subcutaneous xenotransplanted tumor model [37]. circFLNA overexpression has been found to promote tumor growth, inhibit miR-486-3p expression and promote the expressions of XRCC1 and CYP1A1, however, miR-486-3p mimic led to the opposite effect of overexpressed circFLNA. In addition, the effect of circFLNA overexpression on tumor growth could be reversed by miR-486-3p mimic, which further verified the results found in vitro.

The results in this study revealed that circFLNA acted as a sponge of miR-486-3p to promote the proliferation, migration, and invasion of lung cancer cells via regulating XRCC1 and CYP1A1 in vitro and in vivo. It should also be noted that a larger sample size is needed to further reveal the exact mechanism of action of circFLNA in the regulation of miR-486-3p in vivo in lung cancer.

## Supplementary information

checklist
